# Voting and (im)moral behavior

**DOI:** 10.1038/s41598-022-24360-w

**Published:** 2022-12-31

**Authors:** Kajsa Hansson, Emil Persson, Gustav Tinghög

**Affiliations:** 1 Division of Economics, Department of Management and Engineering, Linköping University, 581 83 Linköping, Sweden; 2 Department of Health Medicine and Caring Sciences, The National Center for Priority Setting in Health Care, Linköping University, 581 83 Linköping, Sweden

**Keywords:** Human behaviour, Behavioural methods

## Abstract

Due to diffusion of responsibility, majority voting may induce immoral and selfish behavior because voters are rarely solely responsible for the outcome. Across three behavioral experiments (two preregistered; n = 1983), we test this hypothesis in situations where there is a conflict between morality and material self-interest. Participants were randomly assigned to make decisions about extracting money from a charity either in an experimental referendum or individually. We find no evidence that voting induces immoral behavior. Neither do we find that people self-servingly distort their beliefs about their responsibility for the outcome when they vote. If anything, the results suggest that voting makes people less immoral.

## Introduction

Large societal and global challenges require collective action. In such situations, democratic decision making can play an important role in promoting unselfish behavior among large groups of people. When people make democratic decisions, a pro-social majority can override a selfish minority and thus provide large amounts of resources to foreign aid, redistributive matters, and to social security. But several lines of research suggest that people can act more selfishly if they can share the responsibility with others. Because a democratic outcome is the result of the decisions of the group members, one person is never solely responsible for the outcome. This may lead voters to feel a diffusion of responsibility for bad outcomes, making them act more immoral and selfish when voting than when making equivalent choices individually. Although recent studies have linked diffusion-of-responsibility effects to immoral outcomes in both markets and organizations^1–3^, less attention has been devoted to exploring whether democratic decision-making alters behavior and moral inclination in a similar fashion. This is surprising, given that voting is similar to decisions made in markets with regards to the shared responsibility of the outcome. In this paper, we present a series of incentivized and pre-registered studies testing the hypothesis that voting increases immoral and selfish behavior.

The general literature on group decision making and morality emphasizes “shared guilt” and diffused responsibility as the distinguishing feature of decisions taken in groups^4–8^. People may not care much about the morality of an outcome if they do not feel responsible for its occurrence. This implies that the feeling of guilt for bad outcomes diminishes as the number of people supporting the same outcome increases. As a consequence, people should become more willing to act immoral and self-serving in contexts that facilitate guilt sharing, as opposed to in an individual choice context where they have to bear all the guilt themselves. Recent empirical studies have also provided tentative support for this hypothesis. For example, Falk and Szech^2^ showed that people in groups of eight were more likely to make an immoral choice—earn money for killing a mouse—under a group decision-rule where eight mice were killed if at least one person opted to kill his or her mouse, as opposed to an individual choice condition where people were fully responsible for the life or death of a single mouse. Using the same “mouse-paradigm”, Falk and Szech^1^ also found that the willingness to kill a mouse for money was substantially higher for decisions made in a market situation compared to individual decisions. What these findings seem to suggest is that diffusion of responsibility is a probable mechanism when it comes to understanding when and why immoral behavior arises.

In this study, we investigate diffusion of responsibility in the context of voting. To address this question, we conducted three incentivized behavioral experiments (n = 1982) where choices have consequences for real people. Participants were asked to make a decision between money for themselves or a donation to a charitable cause. Thus, similar to the decisions in e.g. Falk and Szech^1^ these decisions mimic situations in which own gains can be sacrificed to avoid negative consequences for a third party. All experiments had a voting condition and an individual condition. Participants in the voting condition made their decision collectively by voting, while participants in the individual condition made their decisions individually. In Study 1, we explore the influence of voting (simple majority rule) in groups of 49 people, in a situation where people could donate to a potentially lifesaving charitable cause. In Study 2, we study how voting (simple majority rule) in groups of five people affects immoral behavior. Participants could extract money from a potentially lifesaving charitable donation, and we also collected data on people’s beliefs of making a pivotal decision. In Study 3, we follow the same experimental paradigm but explore the influence of voting with a median rule. Our data, materials, and preregistrations can be accessed on the Open Science Framework: https://osf.io/ztvf8/.

## Study 1: Majority voting in large groups

For our first study, we explored if voting affects behavior both in a moral decision and in a morally neutral consumption decision. In the experiment, participants in the voting condition voted on the two decisions in voting groups of 49 people, while people in the individual condition decided individually.

### Methods

In total 574 participants participated in the experiment (52.8% male; mean age = 24.1 years, SD = 4.0). The sample size allowed detection of small-sized effects (h = 0.23) with 80% power. Participants were undergraduate students recruited using the Online Recruitment System for Economic Experiments (ORSEE^9^) at Linköping University, Sweden. Experimental instructions were presented on a computer screen and were programmed in Qualtrics. All methods were carried out in accordance with relevant ethical guidelines and regulations. All decisions were made anonymously. All participants gave informed consent prior to participation. According to guidelines from the Swedish research council concerning the Ethical Review of Research Involving Humans (SFS 2003:460), approval from an ethics committee is not required for behavioral research such as this study.

We used a moral choice paradigm based on the vaccine paradigm in Kirchler et al.^3^ and Sutter et al.^10^. Participants were asked to choose between receiving 63 SEK (approx. $7.5) for themselves and donating 63 SEK to UNICEF (representing 20 doses of measles vaccine). The donation of measles vaccine is a potentially lifesaving choice that can save 10 people’s lives (two doses of measles vaccine are required for full protection). Hence, taking the money imposes a negative externality on children in need. In addition, participants also made a consumer choice, between receiving a thermos mug and receiving 30 SEK (approx. $3.50). This choice was included to serve as a morally neutral decision. The vaccine and thermos paradigms were both fully incentivized and all decisions for all participants were carried out for real. In total, the experiment generated 6840 doses of measles vaccine to UNICEF and 209 thermos mugs to participants.

Each participant was randomly assigned to one of two conditions: The *Voting condition* or the *Individual condition*. In the voting condition participants were randomly grouped into groups consisting of 49 participants. Everything was identical across conditions except that in the voting condition majority rule decided the outcome of the decisions. Hence, while participants in the individual condition were informed that their two decisions were going to be implemented for themselves, participants in the voting condition were informed that the option that the majority chose would be implemented for everyone.

At the end of the experiment, participants stated their age and sex and responded to the short version of the Cultural Cognition World View Group Scale^11^, a 6-item measure that indicates if individuals are more predisposed toward an individualistic or a collectivistic worldview. For example, one of the items asks respondents to state their level of agreement or disagreement with the statement that “The government interferes far too much in our everyday lives.” Responses were made on a four-point Likert scale ranging from 1 (strongly disagree) to 4 (strongly agree). A high score indicates a more individualistic worldview, whereas a low score indicates a more collectivistic worldview. Experimental instructions, data, and code are available at: https://osf.io/ztvf8/.

### Results

To investigate if voting affects immoral behavior, we compare the proportion of participants that took the money for themselves (thus not donating the same amount to UNICEF for the measles vaccine) across the two conditions. In the individual condition, 21.1 percent of the participants chose to take the money for themselves instead of donating to UNICEF, while 21.4 percent did the same when voting (*x*^2^(1) = 0.012, *p* = 0.92, n = 574). Thus, responses were almost identical across conditions, meaning that voting did not significantly change immoral behavior. The results were also similar across conditions for the consumption decision, where 62.5 percent of participants in the individual condition chose to take money instead of the thermos mug, and 64.6 percent did the same thing in the voting condition (*x*^2^(1) = 0.28, *p* = 0.60, n = 574). We test the robustness of these results in a logistic regression framework controlling for sex, age, and worldview, see Table 1. The estimated differences between conditions remain small and insignificant for both the moral decision and the consumption decision. Age and worldview had a small but insignificant impact on decisions. Males were more likely to take the money instead of donating, which is consistent with previous studies showing that males in general are less prosocial in these types of choices^12^.

**Table 1:** Logistic regression for the effect of majority voting on the immoral decision and the consumption decision in Study 1.

	(1)	(2)
	Immoral choice	Consumption choice
Voting condition	0.005	0.015
	(0.034)	(0.040)
Male	0.121***	0.015
	(0.033)	(0.041)
Age	0.002	0.011*
	(0.004)	(0.007)
Worldview	0.010*	− 0.003
	(0.005)	(0.006)
Observations	574	574

Coefficients represent marginal effects (at means), standard errors in parentheses. Dependent variable in (1) is Immoral behavior = 1 if subjects chose 63 SEK for themselves, and = 0 if subjects instead chose donation to UNICEF. Dependent variable in (2) is consumption choice = 1 if subjects chose 30 SEK for themselves, and = 0 if subjects chose the thermos mug. Worldview score is a continuous variable (ranging from 6 to 24), where 6 is the most collectivistic score and 24 is the most individualistic score.

***p < 0.01; **p < 0.05; *p < 0.1.

## Study 2: Majority voting in groups of five

In Study 2, we assigned all participants to groups of five. To investigate the possible relationship between pivotality and immoral behavior we collected data on participants’ beliefs of being pivotal. While Study 1 contained only one moral choice, Study 2 contained a series of four moral choices where participants could extract money from different charitable causes. Moreover, a limitation of Study 1 was that we varied both voting vs. individual choice and belonging to a group or not across conditions. To address this concern, we made group membership salient to subjects in both conditions in Study 2. Hypotheses and main analysis plan were specified and pre-registered before data collection: https://osf.io/ztvf8/.

### Methods

In total, 704 English-speaking participants were recruited from Prolific^13^, to participate in the online experiment (47.6% males; mean age = 33.9 years, SD = 12.6). The sample size allowed us to detect small-sized effects (d = 0.21) with 80% power. The experiment was programmed in Qualtrics. All methods were carried out in accordance with relevant ethical guidelines and regulations. All participants gave informed consent prior to participation. According to guidelines from the Swedish research council concerning the Ethical Review of Research Involving Humans (SFS 2003:460), approval from an ethics committee is not required for behavioral research such as this study.

All participants were assigned to a group of five participants. Each group was entrusted with a donation of 150 tokens (10 tokens = £ 0.5) designated to a charitable cause and participants could choose to extract tokens (for own payment) from the donation. The following two options were presented to participants:

- **Option A.** Extract 0 tokens from the donation.
- **Option B.** Extract 30 tokens from the donation.

Participants were randomly assigned to one of two conditions, such that decisions were made either individually or by majority voting. In the *Majority voting condition*, participants were informed that the option chosen by the majority in the group was going to be implemented for all group members. The choices and the consequences were the same for all five members of the group. In the *Individual condition*, participants made decisions as individual decision makers, and all five members of the group had their own decisions implemented. Thus, group membership was made salient also in the individual condition by informing group members that they were all tapping the same common resource, i.e., the fund of 150 tokens designated for donation. Four different charity organizations were presented, and participants made a binary decision for each organization. One of the four organizations was randomly selected for payment for each group.

In the next stage of the experiment, we measured participants’ beliefs of being pivotal. Participants stated their perceived likelihood (on a scale from 1 to 6) that half of the other group members would choose Option A and the other half would choose Option B, thus making the respondent pivotal. Participants also stated their beliefs about how many of the other group members chose to extract from the donation. Correct answers were monetarily rewarded. Finally, participants responded to individual difference measures related to moral inclinations. Similar to Study 1, participants responded to the Cultural Cognition World View Group Scale^11^ (see Study 1 for description). Since the Cultural Cognition World View Group Scale focuses on peoples’ views on the government’s role to secure collective welfare, we also wanted to control for the degree the importance one places on being a moral person. Therefore, we included the Moral Identity Scale^14^. The scale has two dimensions of moral identity: internalization and symbolization. A higher score on internalization indicates that morality is central to one’s self-concept. A higher score on symbolization reflects that morality is expressed outwardly to others to a high degree. Symbolization is usually understood as the more social aspects of moral identity. The full experimental instructions, data, and code are available at: https://osf.io/ztvf8/.

### Results

**Figure 1:**
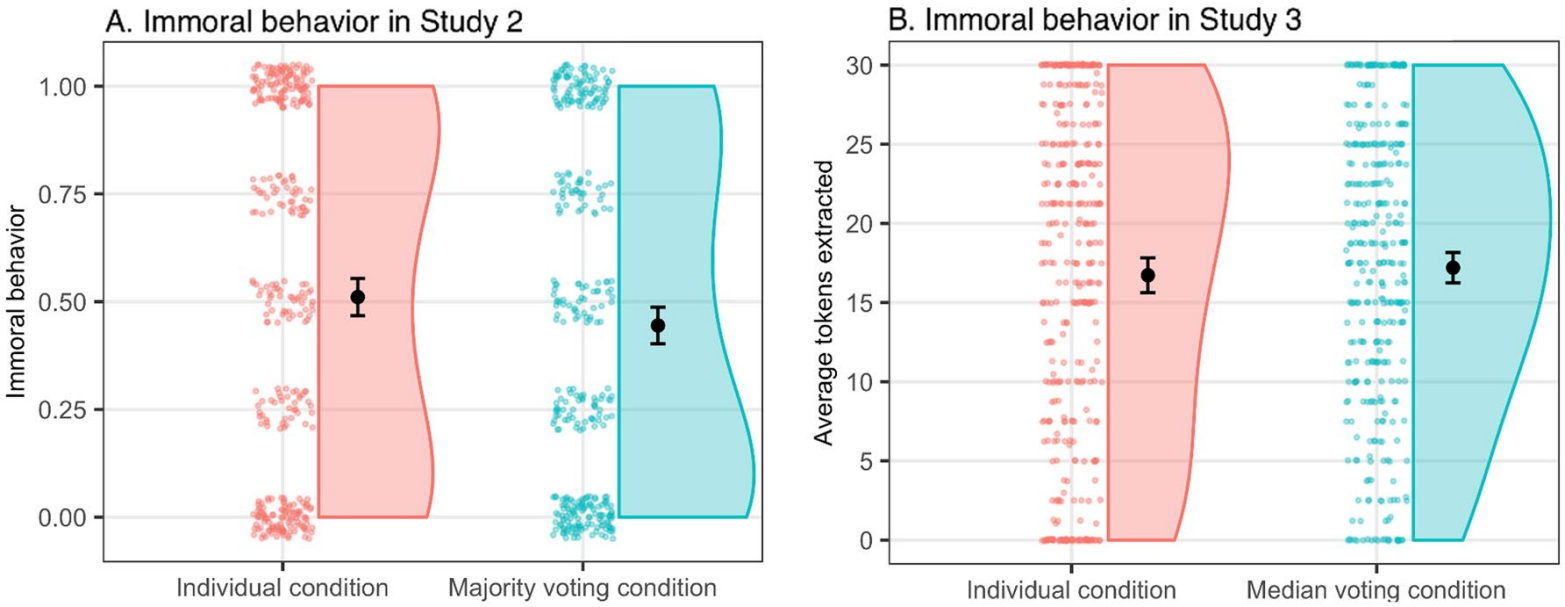
(**A**) Immoral behavior by condition in Study 2 (Majority voting), n = 704. (**B**) Immoral behavior by condition in Study 3 (Median voting), n = 704. Bars indicate 95% confidence intervals.

To investigate if voting affects immoral behavior, we compare extracted tokens from charity across conditions. Specifically, we operationalized immoral behavior as the proportion (for each participant) of the four binary decisions where the participant chose to extract from the donation. Thus, a value of 0 means that the participant chose not to extract from any of the charities, and a value of 1 means that the participant chose to extract tokens from all four charities. Our main confirmatory test of the effect of voting on immoral behavior shows that voting significantly decreased immoral behavior (see Fig. 1A). The average proportion of charities where participants chose to extract from the donation was 0.51 in the individual condition and 0.44 in the voting condition, meaning that the estimated mean difference in immoral behavior between conditions is around six percentage points (*t*(702) = 2.14, *p* = 0.03). The direction of this effect was the same for all four decisions, see Table S1 in Supplementary Material for details.

We follow up with data-contingent analyses to assess the robustness of these findings. Table 2 presents the regression analyses where we controlled for sex, age, worldview, symbolization, and internalization of moral principles. The estimate for the majority voting condition shows that voting significantly decreased immoral behavior by 6.2 percentage points when controlling for sex, age, worldview, and the moral identity scale. Although the regression results show that males extracted significantly more from the donation compared to women, the decrease in immoral behavior in the majority voting condition appears to be driven by males. Majority voting significantly decreased immoral behavior by 18 percentage points for males, while selfish behavior among females remained unaffected (see Table S6 in Supplementary Materials). The regression results also show that internalization of moral principles was associated with less immoral behavior, while symbolization of moral principles and worldview were not significantly correlated with immoral behavior.

**Table 2:** The effect of majority voting (Study 2) and median voting (Study 3) on immoral behavior.

	(1)	(2)	(3)	(4)
	Immoral behavior	Immoral behavior	Immoral behavior	Immoral behavior
Majority voting condition	− 0.061**	− 0.062**		
	(0.031)	(0.031)		
Median voting condition			0.423	0.441
			(0.737)	(0.738)
Male	0.075**	0.059*	2.313***	2.162***
	(0.031)	(0.031)	(0.730)	(0.740)
Age	− 0.000	− 0.001	0.036	0.037
	(0.001)	(0.001)	(0.030)	(0.030)
Worldview	0.006	0.002	0.079	0.022
	(0.018)	(0.018)	(0.461)	(0.466)
Internalization of moral principles		− 0.065***		− 0.452
		(0.019)		(0.465)
Symbolization of moral principles		− 0.002		− 0.010
		(0.014)		(0.355)
Constant	0.465***	0.894***	14.237***	17.217***
	(0.076)	(0.141)	(1.872)	(3.491)
Observations	704	704	704	704

All regressions are ordinary least squares. The dependent variable in model (1) and (2) is the mean value of how much participants extract from the donation across the four moral decisions (0 = do not extract anything from the donations, 1 = extract the full amount of 30 tokens across all four organizations). The dependent variable in model (3) and (4) is the mean value of how much participants extract from the donation across the four moral decisions (0 = do not extract anything from the donations, 30 = extract the full amount of 30 tokens across all four organizations). Worldview score is a continuous variable (ranging from 6 to 24), where 6 is the most collectivistic score and 24 is the most individualistic score. Internalization of moral principles is a continuous variable (ranging from 1 to 7), where a high score indicated that moral principles are central to one’s self-concept. Symbolization of moral principles is a continuous variable (ranging from 1 to 7), where a high score indicated that moral principles are expressed outwardly to others to a high degree.

***p < 0.01; **p < 0.05; *p < 0.1.

**Figure 2:**
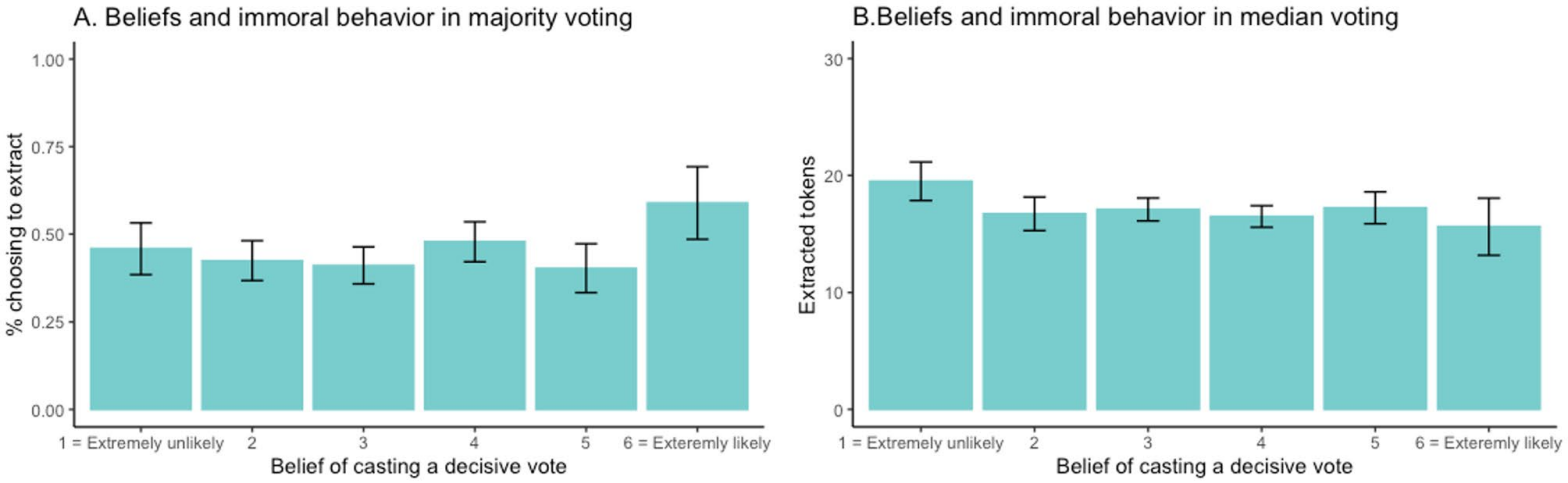
(**A**) Immoral behavior and beliefs of being pivotal in the majority voting condition (Study 2). (**B**) Immoral behavior and beliefs of being pivotal in the median voting condition (Study 3). Bars indicate 95% confidence intervals.

Following the pre-registered analysis plan, we explored the relationship between beliefs of being pivotal and immoral behavior for participants in the majority voting condition. When people feel it is unlikely that their vote will be decisive for the outcome, they may also feel a declining responsibility for the outcome of a vote. Following this line of reasoning, individuals’ beliefs of being pivotal should be associated with immoral behavior. We therefore examined the link between immoral behavior and participants’ belief of the likelihood that their decision was pivotal. Figure 2A illustrates the relationship between immoral behavior and beliefs of being pivotal by presenting the average immoral behavior conditioned on each point on the pivotal scale. The figure shows no clear pattern of decreased immoral behavior with the belief of being pivotal when voting. We test the relationship between immoral behavior and beliefs of being pivotal in a panel regression model (see Supplementary Materials Table S2), which confirms that there is no significant negative relationship between immoral beliefs of being pivotal when voting (β = − 0.01, *t*(352) = − 0.07, *p* = 0.95).

Finally, we test if people distort their beliefs of other people’s behavior in a self-serving manner when they vote. In the majority voting condition, feelings of responsibility for the outcome can be diminished as the (expected) number supporting the immoral outcome is increases. If people want to reduce feelings of responsibility in the majority voting condition, they can increase their expectations of the number supporting the decision to extract from the donation. The data shows that, on average, participants in the majority voting condition expected that 1.75 out of 4 of the other group members would choose to extract from the donation (see Table S8 in Supplementary Materials). In fact, participants in the majority voting condition expected slightly *fewer* group members to extract from the donation, but this difference was not statistically significant (*t*(702) = − 1.80, *p* = 0.07). Thus, although merely suggestive, this indicates that voting may affect expectations of moral behavior, subsequently, people’s tendency to act unselfishly.

## Study 3: Median voting in groups of five

Neither Study 1 nor Study 2 provided any supporting evidence for the hypothesis that immoral behavior increases when outcomes are determined by voting. In Study 3 we use the same experimental setup as in Study 2 but test another voting rule—Median voting^15^.

### Methods

In total 705 English-speaking participants were recruited from Prolific^13^ to participate in an online experiment (46.2% male; Mean age = 33.3 years, SD = 12.3). The sample size allowed detection of small-sized effects (d = 0.21) with 80% power. The experiment was programmed in Qualtrics. All methods were carried out in accordance with relevant ethical guidelines and regulations. All participants gave informed consent prior to participation. Hypotheses and main analysis plan were specified and pre-registered before data collection: https://osf.io/ztvf8/.

Like Study 2, all participants were assigned to a group of five participants. Each group was entrusted with a donation of 150 tokens (10 tokens = £ 0.5) designated to a charitable cause and participants could choose to extract tokens (for own payment) from the donation. Participants were randomly assigned to one of two conditions, such that outcomes were determined either by participants’ individual choices or by a median voting rule. In the *Median voting condition,* each person in the group proposed an amount, between 0 and 30 tokens, to extract from the donation. The median proposal was then implemented for all group members. In the *Individual condition*, participants made decisions as individual decision makers (choosing an amount to extract between 0 and 30 tokens), and all five participants in the group had their own decisions implemented. Participants indicated their beliefs of being pivotal and their beliefs about the other group members’ decisions. Participants stated their perceived likelihood (on a scale from 1 to 6) that their proposal was the median value in the group, and how many group members they expected to extract more/less than themselves. The full experimental instructions, data, and code are available at: https://osf.io/ztvf8/.

### Results

The main result from the experiment is presented in Fig. 1B. In the median voting condition, participants extracted on average 17.20 tokens from the charitable cause, and in the individual condition, they extracted on average 16.72 tokens. Thus, the estimated effect was small and insignificant (mean difference = − 0.48, *t*(703) = − 0.65, *p* = 0.52). Further, we found no significant differences across conditions when testing each of the four decisions separately, see Table S4 in Supplementary Materials for details. Table 2 presents data-contingent analyzes focusing on the effect of median voting on immoral behavior in a regression framework, controlling for sex, age, worldview, and the moral identity scale. The results confirm that median voting did not significantly affect immoral behavior; the point estimate of 0.4 tokens is small and statistically insignificant. Males extracted more from the donations, but none of the other covariates had any significant effect on immoral behavior.

Figure 2B illustrates the relationship between beliefs of being pivotal and immoral behavior by presenting the average extracted amount conditioned on each point on the pivotal scale. As can be seen in Fig. 2B, there was no clear relationship between immoral behavior and beliefs of being pivotal. To test the correlation between beliefs of being pivotal we conducted a panel regression on tokens extracted from the charity organizations controlling for beliefs of being pivotal, gender, and age (see Table S2 in Supplementary Materials). The regression results show that there was no significant correlation between tokens extracted from the donations and beliefs of being pivotal (β = − 0.15, *t*(351) = − 0.71, *p* = 0.48). Lastly, we examine if voting makes people distort their beliefs about their responsibility for the outcome. To do this, we look at participants’ expectations regarding other participants’ behavior (i.e., how much they would extract). If participants in the median condition adjust their expectations about the other group members’ behavior in a self-serving manner, they would expect a higher proportion of participants to extract more than themselves, compared to the individual condition. Because the median decision is implemented in the voting condition, the more group members they expect to extract more than themselves would decrease feelings of responsibility for the outcome*.* We find no support for this hypothesis. Participants in the median voting condition on average believed that 1.31 people would extract a higher amount from the donation than themselves (See Table S9 in Supplementary Materials). These beliefs are not statistically significantly different from the individual condition (*t*(702) = − 0.90, *p* = 0.36). Taken together, these results do not provide any support for diffusion of responsibility.

## Discussion

To test if voting affects immoral behavior we conducted three experiments. Participants made decisions between taking money or donating money either in an experimental referendum or individually. We found no evidence that voting increases selfish and immoral behavior when decisions are made with majority rule (Study 1 and 2), nor with median voting rule (Study 3). On the contrary, we find suggestive evidence that people donate *more* money when outcomes are determined by a majority voting rule (Study 2). Thus, we provide no evidence that voting increases selfish and immoral behavior.

Moreover, we find no evidence supporting the idea that people who vote self-servingly distort their beliefs about their responsibility for the outcome. First, we find no correlation between perceptions of being pivotal and the decision to extract from the donation (Study 2 and Study 3). Second, we do not find that voting makes people expect other group members to act more immoral. Contrary to the predictions from diffusion of responsibility, we find suggestive evidence that majority voting *decreases* the expectations of the other group members to extract from the donation (Study 2). A possible interpretation is that the expectation of acting moral is stronger when people vote, which can explain why voting in some cases may increase moral behavior.

In our experimental designs we went to great lengths to provide conditions favorable to diffusion of responsibility thus giving the hypothesis a maximum chance to get empirically supported. In Study 1 we assigned participants to large groups of 49 people, which is a considerably larger group than what previous literature has used when studying diffusion of responsibility^1,3^. We tested two different types of voting mechanisms, majority voting and median voting. To further explore if moral responsibility is diffused, we controlled for participants’ beliefs of casting a decisive vote using both monetary incentivized and non-incentivized questions. Thus, our findings provide an important extension to previous literature on moral decision-making in markets showing that people are less moral when the responsibility for a moral outcome is diffused through market interaction^1–3,16^.

The fact that people become more immoral in market situations cannot solely be explained by diffusion of responsibility, because then we should see similar results also in the context of democratic decision making. An alternative interpretation considering our results would be that institutional settings affect social norms, which is a main driver of immoral behavior. This interpretation would also be supported by some studies that have examined immoral behavior in markets (e.g.,^17–19^). For example, Bartling and Özedemir^17^ use a competitive environment to study if people in markets use the argument of “If I don’t do it, someone else will” to justify selfish behavior in markets. Their findings suggest that people use a replacement excuse, in the absence of a social norm, to justify selfish behavior. Future studies should explore the boundary conditions for when and why diffusion of responsibility affects immoral behavior.

Our results also contribute to the literature on low-cost theory of expressive voting. The basic idea following Tullock^20^ is that if people believe that their vote is unlikely to be decisive, they are more likely to behave prosocially because they can express a socially good view at a low expected cost. The experimental literature on expressive voting literature has studied how the notion of being pivotal affects voter behavior, typically by manipulating the pivotality of a voter’s decision. The results from the expressive voting literature are mixed^21^. Some studies find little or no support for the theory^22–26^ while other studies find clear indications that expressive voting seems to matter^27,28^. We differ from this literature by studying how voting—in comparison to individual decision-making—affect moral behavior. Since we find that beliefs of being pivotal were not associated with immoral behavior, our study provides no support for low-cost theory of expressive voting at the general level. It should be noted, however, that it is still possible that the two effects are working at the same time but in opposite directions. Some following the arguments of diffusion of responsibility and some acting in line with low cost-theory of expressive voting.

Understanding how voting affects moral behavior is important because it has far-reaching implications for policy outcomes because referendums typically involve conflicts between moral preferences and material self-interest. If voting makes people act more selfishly, it may have detrimental consequences for democratic outcomes and for the provision of the common good. In a series of studies, we found no evidence that voting increases immoral behavior. Instead, our results show that voting in some cases may decrease immoral behavior.

## Supplementary Information

Supplementary Information.

## Data Availability

The study was preregistered at https://osf.io/ztvf8/; data, analysis codes, and Supplementary materials (including experimental instructions and decision screens) can be accessed via https://osf.io/ztvf8/.
