# Supplementary material for: Voting and (im)moral behavior
Source: Sci Rep. 2022 Dec 31;12:22643. doi: 10.1038/s41598-022-24360-w (PMC9805423; doi:10.1038/s41598-022-24360-w)
Supplement: Supplementary file 1 — Supplementary Information. [file 41598_2022_24360_MOESM1_ESM.docx]

# Supplementary Materials

Voting and (Im)moral behavior

Authors: K.Hansson, E.Persson, G.Tinghög

Table of Contents.

1. Additional analyses……………………………………………………………………2
2. Instructions Study 1……………………………………………………………………4
3. Instructions Study 2……………………………………………………………………8
4. Instructions Study 3…………………………………………………………..………30

## Additional analyses

**Table S1.** Proportion choosing Option B (extract 30 tokens) for each organization, by condition

|  | Individual condition | Majority voting condition | P-value (chi2) |
| --- | --- | --- | --- |
| UNICEF | 50.43 % | 43.63 % | 0.07 |
| VI forest | 56.70% | 50.14% | 0.08 |
| World cancer | 48.15% | 39.94% | 0.03 |
| World food programme | 49.00% | 44.19% | 0.20 |
| Observations | 351 | 353 |  |

**Table S2.** Correlation between immoral behavior and belief of being pivotal

|  | (1) | (2) |
| --- | --- | --- |
|  | Immoral behavior: Majority Voting | Immoral behavior: Median voting condition |
|  | Coefficient | Coefficient |
|  |  |  |
| Beliefs of being pivotal | -0.001 | -0.148 |
|  | (0.012) | (0.209) |
| Constant | 0.447*** | 17.676*** |
|  | (0.038) | (0.672) |
|  |  |  |
| Observations | 1,412 | 1,408 |
| Number of ID | 353 | 352 |

**Notes.** All regressions are fixed effect models with standard errors clustered on subject ID. The dependent variable in model (1) is a binary variable where 0=do not extract anything from the donation, 1=extract 30 tokens from the donation. The dependent variable in model (2) is how much participants extract from the donation (0=do not extract anything from the donations, 30=extract the full amount of 30 tokens). *** p<0.01, ** p<0.05, * p<0.1

**Table S3. Logistic regression for the correlation between of beliefs of being pivotal and immoral behavior**

|  | UNICEF | VI Agroforestry | World Child Cancer | World Food Programme |
| --- | --- | --- | --- | --- |
|  | Marginal effect | Marginal effect | Marginal effect | Marginal effect |
|  |  |  |  |  |
| Worldview | -0.008 | -0.036 | -0.005 | 0.012 |
|  | (0.029) | (0.029) | (0.029) | (0.029) |
| Male | -0.023 | -0.090* | 0.031 | 0.022 |
|  | (0.053) | (0.054) | (0.053) | (0.054) |
| Age | 0.001 | -0.002 | -0.001 | 0.001 |
|  | (0.002) | (0.002) | (0.002) | (0.002) |
| Belief Pivotal UNICEF | 0.018 |  |  |  |
|  | (0.019) |  |  |  |
| Belief Pivotal Vi Agroforestry |  | -0.019 |  |  |
|  |  | (0.020) |  |  |
| Belief Pivotal World Child Cancer |  |  | 0.015 |  |
|  |  |  | (0.018) |  |
| Belief Pivotal World Food Programme |  |  |  | 0.027 |
|  |  |  |  | (0.019) |
|  |  |  |  |  |
| Observations | 353 | 353 | 353 | 353 |

**Note:** Presented with marginal effects, standard errors in parentheses. For each organization, dependent variable = 1 if subjects chose to extract 30 tokens from the donation (option B) and = 0 if subjects choose to not extract from the donation (Option A). Beliefs of being pivotal is measured on a six-point scale where 1=extremely unlikely of being pivotal and 6=extremely likely of being pivotal. *** p<0.01, ** p<0.05, * p<0.1

**Table S4.** Tokens extracted for each organization, by condition

|  | Individual condition | Median voting condition | | P-value (t-test) | | P-value (Mann-whitney) | |  |
| --- | --- | --- | --- | --- | --- | --- | --- | --- |
| UNICEF | 16.78 | 16.97 | | 0.81 | | 0.94 | |  |
| VI forest | 16.69 | 16.86 | | 0.82 | | 0.99 | |  |
| World cancer | 16.98 | 17.62 | | 0.44 | | 0.68 | |  |
| World food programme | 16.45 | 17.35 | | 0.29 | | 0.43 | |  |
| Observations | 353 | 352 | |  | |  | |  |
|  |  | |  | |  | |  | |

**Table S5.** The interaction effect of voting and gender on immoral behavior.

|  | (1) | (2) |
| --- | --- | --- |
|  | Moral decision | Consumption decision |
|  |  |  |
| Voting condition | 0.014 | -0.017 |
|  | (0.044) | (0.061) |
| Male | 0.129*** | -0.014 |
|  | (0.047) | (0.058) |
| Voting condition #Male | -0.015 | 0.059 |
|  | (0.066) | (0.081) |
| Age | 0.002 | 0.010* |
|  | (0.004) | (0.005) |
| Worldview | 0.010* | -0.003 |
|  | (0.006) | (0.006) |
| Constant | -0.066 | 0.449*** |
|  | (0.140) | (0.159) |
|  |  |  |
| Observations | 574 | 574 |
| R-squared | 0.031 | 0.009 |

**Note:** All regressions are linear probability models. Dependent variable in (1) is Immoral behavior = 1 if subjects chose 63 SEK for themselves, and = 0 if subjects instead chose donation to UNICEF. Dependent variable in (2) is consumption choice =1 if subjects chose 30 SEK for themselves, and = 0 if subjects chose the thermos mug. Worldview score is a continuous variable (ranging from 6 to 24), where 6 is the most collectivistic score and 24 is the most individualistic score. Robust standard errors in parentheses, *** p<0.01, ** p<0.05, * p<0.1

**Table S6.** The interaction effect of majority voting and gender on immoral behavior.

|  | (1) | (2) |
| --- | --- | --- |
|  | Immoral behavior | Immoral behavior |
|  |  |  |
| Majority voting condition | 0.025 | 0.022 |
|  | (0.042) | (0.042) |
| Male | 0.166*** | 0.148*** |
|  | (0.043) | (0.044) |
| Majority voting condition ×Male | -0.181*** | -0.178*** |
|  | (0.061) | (0.061) |
| Age |  | -0.001 |
|  |  | (0.001) |
| Worldview |  | 0.004 |
|  |  | (0.017) |
| Internalization of moral principles |  | -0.064*** |
|  |  | (0.019) |
| Symbolization of moral principles |  | -0.003 |
|  |  | (0.014) |
| Constant | 0.427*** | 0.837*** |
|  | (0.031) | (0.142) |
|  |  |  |
| Observations | 704 | 704 |
| R-squared | 0.027 | 0.046 |

**Notes.** All regressions are ordinary least squares. The dependent variable is the mean value of how much participants extract from the donation across the four moral decisions (0=do not extract anything from the donations, 1=extract the full amount of 30 tokens across all four organizations). Worldview score is a continuous variable (ranging from 6 to 24), where 6 is the most collectivistic score and 24 is the most individualistic score. Internalization of moral principles is a continuous variable (ranging from 1 to 7), where a high score indicated that moral principles are central to one’s self-concept. Symbolization of moral principles is a continuous variable (ranging from 1 to 7), where a high score indicated that moral principles are expressed outwardly to others to a high degree.Robust standard errors in parentheses, *** p<0.01, ** p<0.05, * p<0.1

**Table S7.** The interaction effect of median voting and gender on immoral behavior.

|  | (1) | (2) |
| --- | --- | --- |
|  | Immoral behavior | Immoral behavior |
|  |  |  |
| Median voting condition | 0.235 | 0.243 |
|  | (1.049) | (1.056) |
| Male | 2.136* | 1.945* |
|  | (1.103) | (1.108) |
| Median voting condition ×Male | 0.384 | 0.430 |
|  | (1.461) | (1.466) |
| Age |  | 0.037 |
|  |  | (0.030) |
| Worldview |  | 0.028 |
|  |  | (0.466) |
| Internalization of moral principles |  | -0.454 |
|  |  | (0.465) |
| Symbolization of moral principles |  | -0.015 |
|  |  | (0.357) |
| Constant | 15.773*** | 17.333*** |
|  | (0.773) | (3.514) |
|  |  |  |
| Observations | 704 | 704 |
| R-squared | 0.015 | 0.018 |

**Note:** All regressions are linear probability models. Dependent variable in (1) is immoral behavior = 1 if subjects chose 63 SEK for themselves, and = 0 if subjects instead chose donation to UNICEF. Dependent variable in (2) is consumption choice =1 if subjects chose 30 SEK for themselves, and = 0 if subjects chose the thermos mug. Worldview score is a continuous variable (ranging from 6 to 24), where 6 is the most collectivistic score and 24 is the most individualistic score. Internalization of moral principles is a continuous variable (ranging from 1 to 7), where a high score indicated that moral principles are central to one’s self-concept. Symbolization of moral principles is a continuous variable (ranging from 1 to 7), where a high score indicated that moral principles are expressed outwardly to others to a high degree. Robust standard errors in parentheses, *** p<0.01, ** p<0.05, * p<0.1

**Table S8.** Expected number of group members choosing to extract from the donation across conditions (majority voting)

|  | Individual condition | Majority voting condition | P-value (t-test) |
| --- | --- | --- | --- |
| Expected number of group members choosing to extract from the donation | 1.89 | 1.75 | 0.07 |
| Observations | 351 | 353 |  |

**Table S9.** Expected number of group members choosing to extract from the donation across conditions (median voting)

|  | Individual condition | Median voting condition | P-value (t-test) |
| --- | --- | --- | --- |
| Expected number of group members choosing to extract more than themselves from the donation | 1.25 | 1.31 | 0.36 |
| Observations | 353 | 351 |  |

### Experimental instructions (Study 1).

In the following we provide an English translation of the instructions for the voting condition and the individual condition.

***The voting condition***

Welcome!

Fill in your ID number from the note located beside your computer. This is important for us to be able to give you the right amount of money based on your answers to the questions. It is important that you save the note with your ID number. The note is the receipt that you will have to show in order to get your compensation.

[NEW SCREEN]

**General information**

- During the study, you are not allowed to talk to other participants and the use of cell phones is not permitted.
- According to economic research practice, all instructions and statements in this study are true.
- All your answers are anonymous, neither experiment leaders nor other participants will know what choices you have made.
- After you have answered the questions and proceeded in the questionnaire, you will not be able to go back and change your decisions.
- Information about how you receive your compensation is stated on the note with your ID number.

[NEW SCREEN]

**Overview of the study**

The study contains three blocks. Before each part, you will receive instructions and it is important that you read them carefully. It is important that you understand how each part works before you begin a new part. If you have any questions, raise your hand and ask the experiment leaders. When you have responded to all questions and have completed the study, please remain seated. The experiment leaders will let you know when you can leave the room.

[NEW SCREEN]

You are part of a group of 49 persons in total. The participants in your group will conduct the study at different times. No one in your group will know who the other participants are; not during, nor after the experiment. You, and the other group participants will vote on a number of issues and decide on the alternative that you consider is the best. The outcome will be decided by majority rule; the alternative most people vote for will be implemented for you and the other participants in your group.

[NEW SCREEN]

**PART 1**

In this part, you will vote on two issues, on which you can vote for alternative A or alternative B for each issue. Remember, the alternative most participants in your group votes for will be implemented for you and the participants of your group.

[NEW SCREEN]

**Information about measles vaccine**

**Measles is a highly infectious and deadly disease. Each day hundreds of children become victims of this disease. The survivors often suffer consequences for their entire lives, such as blindness or brain damage. This occurs even though it is easy to protect children.**

Measles is extremely infectious and spreads especially fast when many people live close together, as in refugee camps. Especially for weakened children, the disease often results in death or leads to lasting physical or mental damage. Measles is one of the main causes of blindness among children and often becomes critical when no medical help is available. This occurs even though measles vaccination offers quick, reliable, and inexpensive protection. UNICEF conducts major vaccination campaigns, especially after natural disasters and in other emergency situations, to prevent the spreading of the disease. Giving measles vaccines not only protects the vaccinated children, it also reduces the risk of contamination for those who come in contact with them.


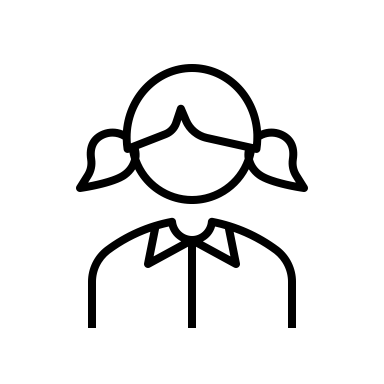
(Note: original image of child used in experiment replaced with placeholder here)

[NEW SCREEN]

**Question 1**

You can either vote for alternative A (you and the other participants in the group receive 63 SEK each) or alternative B (63 SEK for each participant in the group is donated to UNICEF for measles vaccine). A donation of 63 SEK corresponds to 20 doses of measles vaccine. This means that if the majority votes for alternative A, you and the rest of the group receive a sum of money but vaccines will not be sent to children in need. If the majority votes for alternative B, vaccines will be sent to children in need, however, no one in the group will receive money.

Do you vote for alternative A or alternative B?

**Alternative A:** You and the other participants in your group receive 63 SEK per person

**Alternative B:** 63 SEK per person is donated to UNICEF for the measles vaccines

[NEW SCREEN]

**Question 2**

You can either vote for alternative A (you and the other participants in the group receive 30 SEK each) or alternative B (you and the other participants in the group receive a thermos mug). The thermos is shown in the image below. This means that if the majority votes for alternative A, you and the rest of the participants in your group receive a sum of money but no one receives a thermos mug. If the majority votes for alternative B, you and the rest of your group receive a thermos mug each, however, no one in the group will receive money.


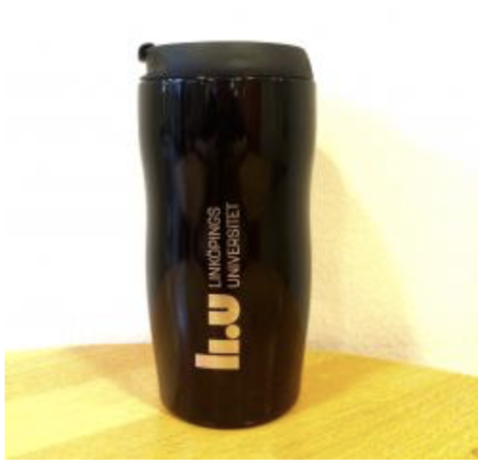


Do you vote for alternative A or alternative B?

**Alternative A:** You and the other participants in your group receive 30 SEK per person

**Alternative B:** You and the other participants in your group receive a thermos mug each

***The individual condition***

Welcome!

Fill in your ID number from the note located beside your computer. This is important for us to be able to give you the right amount of money based on your answers to the questions. It is important that you save the note with your ID number. The note is the receipt that you will have to show in order to get your compensation.

[NEW SCREEN]

**General information**

- During the study, you are not allowed to talk to other participants and the use of cell phones is not permitted.
- According to economic research practice, all instructions and statements in this study are true.
- All your answers are anonymous, neither experiment leaders nor other participants will know what choices you have made.
- After you have answered the questions and proceeded in the questionnaire, you will not be able to go back and change your decisions.
- Information about how you receive your compensation is stated on the note with your ID number.

[NEW SCREEN]

**Overview of the study**

The study contains three blocks. Before each part, you will receive instructions and it is important that you read them carefully. It is important that you understand how each part works before you begin a new part. If you have any questions, raise your hand and ask the experiment leaders. When you have responded to all questions and have completed the study, please remain seated. The experiment leaders will let you know when you can leave the room.

[NEW SCREEN]

**PART 1**

In this part, you will respond to two questions where you can choose alternative A or alternative B in each question. The alternative you choose in both questions will be implemented for you.

[NEW SCREEN]

**Information about measles vaccine**

**Measles is a highly infectious and deadly disease. Each day hundreds of children become victims of this disease. The survivors often suffer consequences for their entire lives, such as blindness or brain damage. This occurs even though it is easy to protect children.**

Measles is extremely infectious and spreads especially fast when many people live close together, as in refugee camps. Especially for weakened children, the disease often results in death or leads to lasting physical or mental damage. Measles is one of the main causes of blindness among children and often becomes critical when no medical help is available. This occurs even though measles vaccination offers quick, reliable, and inexpensive protection. UNICEF conducts major vaccination campaigns, especially after natural disasters and in other emergency situations, to prevent the spreading of the disease. Giving measles vaccines not only protects the vaccinated children, it also reduces the risk of contamination for those who come in contact with them.


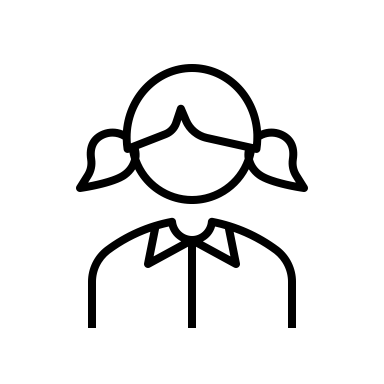
(Note: original image of child used in experiment replaced with placeholder here)

[NEW SCREEN]

**Question 1**

You can choose either alternative A (you receive 63 SEK) or alternative B (63 SEK is donated to UNICEF for the measles vaccine). A donation of 63 SEK corresponds to 20 doses of measles vaccine. This means that if you choose alternative A, you receive a sum of money but the vaccine will not be sent to children in need. If you choose alternative B, the vaccine will be sent to children in need, however, you will not receive any money.

Do you choose alternative A or alternative B?

**Alternative A:** You receive 63 SEK

**Alternative B:** 63 SEK is donated to UNICEF for the measles vaccine

[NEW SCREEN]

**Question 2**

You can choose either alternative A (you receive 30 SEK) or alternative B (you receive a thermos mug). The thermos mug is shown in the image below. This means that if you choose alternative A, you receive a sum of money but you do not receive a thermos mug. If you choose alternative B, you receive a thermos mug, however, you will not receive money.


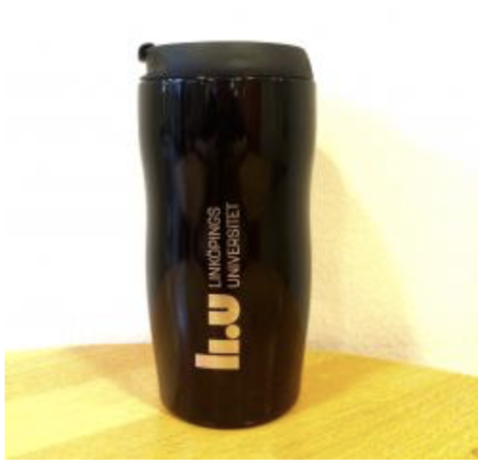


Do you choose alternative A or alternative B?

**Alternative A:** You receive 30 SEK

**Alternative B:** You receive a thermos mug

### Experimental instructions (Study 2)

**INSTRUCTIONS PART 1**

*MAJORITY VOTING CONDITION*

Welcome and thank you for your participation in this study!
   
 **All statements made in this study are true i.e there is no deception in this study.**

During this study we will speak in terms of tokens instead of pounds (GBP). Your earnings will be calculated in terms of tokens and then translated at the end of the study into pounds (GBP) at the following rate:

**10 tokens = £ 0.5**

You will receive a participation fee (simply for completing this study). In addition, you have the possibility to gain additional earnings.

**Please note:** During the study, there will be an attention check in order to ensure that you are paying attention to the study instructions. If you fail this attention check you will be excluded from the study, and you will receive no payment.

Participation in the study should take about 14 minutes. Your decisions in this study will not be known to any other participant, i.e., your anonymity is ensured. There are no foreseeable risks to your participation in this study. By clicking on the button below, you indicate that you understand the information that was presented and that your participation is voluntary, and you may withdraw your consent and discontinue participation in the project at any time.

- I consent to participate in this study

Please enter your Prolific ID

________________________________________________________________

 [NEW SCREEN]

**Your group.**

In this study, you are together with 4 other participants from Prolific i.e. in a group of 5 people. Your group members have been allotted to you at the beginning of the study. You will at no point learn which participant is in your group. 

**The donation.**
Your group is entrusted with a donation of 150 tokens designated to a charitable cause. You and the other people in your group will all be presented with identical questions. Your own earnings and the donation depend on how you and the others respond.

**YOUR DECISION:**

Each person in your group chooses between Option A and Option B:

Option A: Extract 0 tokens from the donation

Option B: Extract 30 tokens from the donation

The option that the majority choose is implemented for all group members. The choices and the consequences are the same for all 5 participants in your group.

**Option A.** If the majority of the group members choose Option A, besides the participation fee you will receive **no additional** money at the end of the study. This holds for all group members: If the majority of the group member choose Option A, no one of the group members will receive additional money.

**Option B.** If the majority of the group members choose Option B, you will **additionally** receive 30 tokens at the end of the study. This holds for all group members: If the majority of the group members choose Option B, all group members will additionally receive 30 tokens. As a further consequence, if the majority of the participants choose option B, 150 tokens of the donation of 150 tokens will be destroyed.

[NEW SCREEN]

Here are two examples:

**Example 1.** The five participants in a group choose:

Participant 1: Option A

Participant 2: Option A

Participant 3: Option B

Participant 4: Option B

Participant 5: Option B

The majority chose Option B. All participants in this group will **additionally** receive 30 tokens per person. As a further consequence, 150 tokens (30*5) of the donation of 150 tokens will be destroyed.

**Example 2.** The five participants in a group choose:

Participant 1: Option B

Participant 2: Option B

Participant 3: Option A

Participant 4: Option A

Participant 5: Option A

The majority chose Option A. Participants in this group receive **no additional money** at the end of the study.

To make sure you understand the instructions, please answer the comprehension questions below.

YOU MUST ANSWER THIS QUESTIONS CORRECTLY TO BE ELIGIBLE TO RECEIVE YOUR ADDITIONAL EARNINGS.

In this study you choose between Option A and Option B:

Option A: Extract 0 tokens from the donation

Option B: Extract 30 tokens from the donation

Suppose two of the group members choose Option B, two group members choose A, and you choose Option A:

1. How many additional tokens do you receive?

- **0 tokens**
- 150 tokens
- 30 tokens
- 60 tokens
- 90 tokens

1. How many tokens of the donation of 150 tokens will be destroyed?

- **0 tokens**
- 150 tokens
- 30 tokens
- 60 tokens
- 90 tokens

Suppose two of the group members choose Option B, two group members choose A, and you choose Option B:

a) How many additional tokens do you receive?

- 0 tokens
- 150 tokens
- **30 tokens**
- 60 tokens
- 90 tokens

b) How many tokens of the donation of 150 tokens will be destroyed?

- 0 tokens
- **150 tokens**
- 30 tokens
- 60 tokens
- 90 tokens

This is an attention check. This test is simple, when asked for a number to enter you must tick on the number “5” below.   Based on the text you read above, what number have you been asked to enter?

- 1
- 2
- 3
- 4
- **5**

*[Correct answers are marked with bold text in this document]*

[NEW SCREEN]

In what follows, four different charity organisations will be presented. You and the other group members will make a decision for each of the four charity organisation. At the end of the study, one of the four organisations will be randomly selected for payment. The decision made by the majority of your group for that particular organisation will be implemented.

 [NEW SCREEN]

**Measles is highly infectious and very often deadly. Each day hundreds of children become victims of this disease. The survivors often suffer consequences for their whole life, like blindness or brain damages. However, protecting the children from measles is easy. Measles vaccination offers quick, reliable, and cheap protection.**

Measles are extremely infectious and spread especially fast when many people live densely together, as in refugee camps. Especially with weakened children the disease often ends deadly or leads to lasting physical or mental damages. Measles are one of the main causes for blindness among children and often become critical when no medical help is available. UNICEF conducts major vaccination campaigns, especially after natural disasters and in other emergency situations, to prevent the spreading of the disease. With a measles vaccination you do not only protect the children, but you also reduce the risk for all who get in contact with them.

[Picture from UNICEF]

[NEW SCREEN]

 [NEW SCREEN]

**YOUR DECISION:**

Your group is entrusted with a donation totaling 150 tokens, corresponding to 30 doses of measles vaccine.

You will now be asked to make a choice between Option A and Option B:

Option A: Extract 0 tokens from the donation to UNICEF

Option B: Extract 30 tokens from the donation to UNICEF

**Remember!** Each person in your group chooses between Option A and Option B. If this decision is selected for payment, the option chosen by the majority will be implemented for all group members.

Please choose between Option A and Option B.

- Option A: Extract 0 tokens from the donation to UNICEF
- Option B: Extract 30 tokens from the donation to UNICEF

 [NEW SCREEN]

**Climate change is hitting hardest on poor farmers in eastern Africa - people who already live on small margins. Through tree planting and agroforestry, farmers can continue to develop their agriculture, despite drought and flooding, and the method ensures families' access to food.**

Donations to the VI Agroforestry contributes to the planting of trees, which helps to reduce poverty and improve the environment in East Africa. Vi Agroforestry work together with small-holder farming families and farmers’ organisations in the Lake Victoria basin in East Africa. The foundation of Vi Agroforestry’s work is sustainable agriculture and agroforestry – growing trees alongside crops and livestock. It provides increased access to food, access to sustainable energy sources and more income.

[Picture from Vi Agroforestry]

[NEW SCREEN]

**YOUR DECISION:**

Your group is entrusted with a donation totaling 150 tokens, corresponding to implantation of five trees.

You will now be asked to make a choice between Option A and Option B:

Option A: Extract 0 tokens from the donation to VI Agroforestry
Option B: Extract 30 tokens from the donation to VI Agroforestry

**Remember!** Each person in your group chooses between Option A and Option B. If this decision is selected for payment, the option chosen by the majority will be implemented for all group members.

Please choose between Option A and Option B.

- Option A: Extract 0 tokens from the donation to VI Agroforestry
- Option B: Extract 30 tokens from the donation to VI Agroforestry

[NEW SCREEN]

**Over 300,000 children worldwide develop cancer each year. If the disease is detected early enough, many of these children can be cured -  80% of children with cancer in the high income world survive. However, in low and middle income countries, survival rates can be as low as 10%, with many children dying without effective pain relief. A reason for this inequality is that many children remain undiagnosed, or are diagnosed too late to be cured, due to a shortage of trained doctors and nurses in-country.**

World Child Cancer work to improve diagnosis, access to treatment and quality of support for children with cancer, and their families, in the low- and middle-income world.  Donations to World Child Cancer supports:

- Chemotherapy for children with cancer
- Pain relief for children with cancer
- Accurate diagnosis of children with cancer

[Picture from WCC]

 [NEW SCREEN]

YOUR DECISION:

Your group is entrusted with a donation totaling 150 tokens, which can support the accurate diagnosis of 4 children with cancer.

You will now be asked to make a choice between Option A and Option B:

Option A: Extract 0 tokens from the donation to World Child Cancer

Option B: Extract 30 tokens from the donation to World Child Cancer

**Remember!** Each person in your group chooses between Option A and Option B. If this decision is selected for payment, the option chosen by the majority will be implemented for all group members.

Please choose between Option A and Option B.

- Option A: Extract 0 tokens from the donation to World Child Cancer
- Option B: Extract 30 tokens from the donation to World Child Cancer

[NEW SCREEN]

**Some 20 million people face catastrophe in South Sudan, Somalia, Nigeria and Yemen with famine already declared in South Sudan. People are already dying from starvation and in South Sudan alone 1 million children are estimated to be acutely malnourished. If we don’t reach people with urgent food aid soon, many of them will die.**

World Food Programme (WFP) is the food-assistance branch of the United Nations and works to help people who cannot produce or obtain enough food for themselves and their families. WFP’s efforts focus on emergency assistance, relief and rehabilitation, development aid and special operations. In emergencies, WFP is often first on the scene, providing food assistance to the victims of war, civil conflict, drought, floods, earthquakes, hurricanes, crop failures and natural disasters.

[Picture from WFP]

 [NEW SCREEN]

**YOUR DECISION:**

Your group is entrusted with a donation totaling 150 tokens, which represents 20 days supply of food to a hungry child.

You will now be asked to make a choice between Option A and Option B:

Option A: Extract 0 tokens from the donation to World Food Programme
Option B: Extract 30 tokens from the donation to World Food Programe

**Remember!** Each person in your group chooses between Option A and Option B. If this decision is selected for payment, the option chosen by the majority will be implemented for all group members.

Please choose between Option A and Option B.

- Option A: Extract 0 tokens from the donation to World Food Programme
- Option B: Extract 30 tokens from the donation to World Food Programme

**INSTRUCTIONS PART 1**

*INDIVIDUAL CONDITION*

Welcome and thank you for your participation in this study!
   
 **All statements made in this study are true i.e there is no deception in this study.**

During this study we will speak in terms of tokens instead of pounds (GBP). Your earnings will be calculated in terms of tokens and then translated at the end of the study into pounds (GBP) at the following rate:

**10 tokens = £ 0.5**

You will receive a participation fee (simply for completing this study). In addition, you have the possibility to gain additional earnings.

**Please note:** During the study, there will be an attention check in order to ensure that you are paying attention to the study instructions. If you fail this attention check you will be excluded from the study, and you will receive no payment.

Participation in the study should take about 15 minutes. Your decisions in this study will not be known to any other participant, i.e., your anonymity is ensured. There are no foreseeable risks to your participation in this study. By clicking on the button below, you indicate that you understand the information that was presented and that your participation is voluntary, and you may withdraw your consent and discontinue participation in the project at any time.

- I consent to participate in this study

Please enter your Prolific ID

________________________________________________________________

[NEW SCREEN]

**Your group.**

In this study, you are together with 4 other participants from Prolific i.e. in a group of 5 people. Your group members have been allotted to you at the beginning of the study. You will at no point learn which participant is in your group.

**The donation.** Your group is entrusted with a donation of 150 tokens designated to a charitable cause. You and the other people in your group will all be presented with identical questions. Your own earnings and the donation depend on how you respond.

**YOUR DECISION:**

Each person in your group chooses between Option A and Option B.

Option A: Extract 0 tokens from the donation

Option B: Extract 30 tokens from the donation

The option that you choose is implemented for you.

Each person in your group will have their own decision implemented.

**Option A.** If you choose Option A, besides the participation fee you will receive **no** **additional** money at the end of the study. This holds for all group members: All group members who choose Option A will receive no additional money.

**Option B.** If you choose Option B, you will **additionally** receive 30 tokens at the end of the study. This holds for all group members: All group members who choose Option B, will additionally receive 30 tokens.

As a further consequence, if you choose Option B, 30 tokens of the donation of 150 tokens will be destroyed. This holds for all group members: All group members who choose Option B, will destroy 30 tokens of the donation.

[NEW SCREEN]

Here are two examples:

**Example 1.** The five participants in a group choose:

Participant 1: Option A

Participant 2: Option A

Participant 3: Option B

Participant 4: Option B

Participant 5: Option B

Participant 3,4 and 5 who chose Option B will **additionally** receive 30 tokens per person. As a further consequence, 90 tokens (30*3) of the donation of 150 tokens will be destroyed. Participant 1 and 2 will receive no additional money.

**Example 2.** The five participants in a group choose:

Participant 1: Option B

Participant 2: Option B

Participant 3: Option A

Participant 4: Option A

Participant 5: Option A

Participant 3,4 and 5 who chose Option A will receive **no additional** money at the ends of the study. Participant 1 and 2 will additionally receive 30 tokens per person at the end of the study. As a further consequence, 60 tokens (30*2) of the donation of 150 tokens will be destroyed.

To make sure you understand the instructions, please answer the comprehension questions below.

YOU MUST ANSWER THIS QUESTIONS CORRECTLY TO BE ELIGIBLE TO RECEIVE YOUR ADDITIONAL EARNINGS.

In this study you choose between Option A and Option B:

Option A: Extract 0 tokens from the donation

Option B: Extract 30 tokens from the donation

Suppose two of the group members choose Option B, two group members choose A, and you choose Option A:

a) How many additional tokens do you receive?

- **0 tokens**
- 150 tokens
- 30 tokens
- 60 tokens
- 90 tokens

b) How many tokens of the donation of 150 tokens will be destroyed?

- 0 tokens
- 150 tokens
- 30 tokens
- **60 tokens**
- 90 tokens

Suppose two of the group members choose Option B, two group members choose A, and you choose Option B:

1. How many additional tokens do you receive?
   - 0 tokens
   - 150 tokens
   - **30 tokens**
   - 60 tokens
   - 90 tokens
2. How many tokens of the donation of 150 tokens will be destroyed?

- 0 tokens
- 150 tokens
- 30 tokens
- 60 tokens
- **90 tokens**

This is an attention check. This test is simple, when asked for a number to enter you must tick on the number “5” below.   Based on the text you read above, what number have you been asked to enter?

- 1
- 2
- 3
- 4
- **5**

*[Correct answers are marked with bold text in this document]*

[NEW SCREEN]

In what follows, four different charity organisations will be presented. You and the other group members will make a decision for each of the four charity organisation. At the end of the study, one of the four organisations will be randomly selected for payment. The decisions you made for that particular organisation will be implemented. The other group members will also have their decision implemented for that particular organisation.

[NEW SCREEN]

**UNICEF**

**Measles is highly infectious and very often deadly. Each day hundreds of children become victims of this disease. The survivors often suffer consequences for their whole life, like blindness or brain damages. However, protecting the children from measles is easy. Measles vaccination offers quick, reliable, and cheap protection.**

Measles are extremely infectious and spread especially fast when many people live densely together, as in refugee camps. Especially with weakened children the disease often ends deadly or leads to lasting physical or mental damages. Measles are one of the main causes for blindness among children and often become critical when no medical help is available. UNICEF conducts major vaccination campaigns, especially after natural disasters and in other emergency situations, to prevent the spreading of the disease. With a measles vaccination you do not only protect the children, but you also reduce the risk for all who get in contact with them.

[Picture from UNICEF]

[NEW SCREEN]

**YOUR DECISION:**

Your group is entrusted with a donation totaling 150 tokens, corresponding to 30 doses of measles vaccine.

You will now be asked to make a choice between Option A and Option B:

Option A: Extract 0 tokens from the donation to UNICEF

Option B: Extract 30 tokens from the donation to UNICEF

**Remember!** Each person in your group chooses between Option A and Option B. If this decision is selected for payment, the option chosen by you will be implemented for you. This holds for all group members: the option that each group member chose in this decision, will be implemented for themselves.

Please choose between Option A and Option B.

- Option A: Extract 0 tokens from the donation to UNICEF (1)
- Option B: Extract 30 tokens from the donation to UNICEF (2)

[NEW SCREEN]

**VI Agroforestry**

**Climate change is hitting hardest on poor farmers in eastern Africa – people who already live on small margins. Through tree planting and agroforestry, farmers can continue to develop their agriculture, despite drought and flooding, and the method ensures families’ access to food.**

Donations to the VI Agroforestry contributes to the planting of trees, which helps to reduce poverty and improve the environment in East Africa. Vi Agroforestry work together with small-holder farming families and farmers’ organisations in the Lake Victoria basin in East Africa. The foundation of Vi Agroforestry’s work is sustainable agriculture and agroforestry – growing trees alongside crops and livestock. It provides increased access to food, access to sustainable energy sources and more income.

[Picture from VI Agroforestry]

[NEW SCREEN]

**YOUR DECISION:**

Your group is entrusted with a donation totalling 150 tokens, corresponding to implantation of five trees.

You will now be asked to make a choice between Option A and Option B:

Option A: Extract 0 tokens from the donation to VI Agroforestry

Option B: Extract 30 tokens from the donation to VI Agroforestry

**Remember!**

Each person in your group chooses between Option A and Option B. If this decision is selected for payment, the option chosen by you will be implemented for you. This holds for all group members: the option that each group member chose in this decision, will be implemented for themselves.

Please choose between Option A and Option B.

- Option A: Extract 0 tokens from the donation to VI Agroforestry
- Option B: Extract 30 tokens from the donation to VI Agroforestry

[NEW SCREEN]

**World Child Cancer

 Over 300,000 children worldwide develop cancer each year. If the disease is detected early enough, many of these children can be cured -  80% of children with cancer in the high income world survive. However, in low and middle income countries, survival rates can be as low as 10%, with many children dying without effective pain relief. A reason for this inequality is that many children remain undiagnosed, or are diagnosed too late to be cured, due to a shortage of trained doctors and nurses in-country.**

World Child Cancer work to improve diagnosis, access to treatment and quality of support for children with cancer, and their families, in the low- and middle-income world.  Donations to World Child Cancer supports:

·       Chemotherapy for children with cancer
 ·       Pain relief for children with cancer
 ·       Accurate diagnosis of children with cancer.

[Picture from WCC]

[NEW SCREEN]

**YOUR DECISION:**

Your group is entrusted with a donation totaling 150 tokens, which can support the accurate diagnosis of 4 children with cancer.

You will now be asked to make a choice between Option A and Option B:

Option A: Extract 0 tokens from the donation to World Child Cancer

Option B: Extract 30 tokens from the donation to World Child Cancer

**Remember!**

Each person in your group chooses between Option A and Option B. If this decision is selected for payment, the option chosen by you will be implemented for you. This holds for all group members: the option that each group member chose in this decision, will be implemented for themselves.   Please choose between Option A and Option B.

- Option A: Extract 0 tokens from the donation to World Child Cancer
- Option B: Extract 30 tokens from the donation to World Child Cancer

[NEW SCREEN]

**World Food Programme**

**Some 20 million people face catastrophe in South Sudan, Somalia, Nigeria and Yemen with famine already declared in South Sudan. People are already dying from starvation and in South Sudan alone 1 million children are estimated to be acutely malnourished. If we don’t reach people with urgent food aid soon, many of them will die.**

World Food Programme (WFP) is the food-assistance branch of the United Nations and works to help people who cannot produce or obtain enough food for themselves and their families. WFP’s efforts focus on emergency assistance, relief and rehabilitation, development aid and special operations. In emergencies, WFP is often first on the scene, providing food assistance to the victims of war, civil conflict, drought, floods, earthquakes, hurricanes, crop failures and natural disasters.

[Picture from WFP]

[NEW SCREEN]

**YOUR DECISION:**

Your group is entrusted with a donation totaling 150 tokens, which represents 20 days supply of food to a hungry child.

You will now be asked to make a choice between Option A and Option B:

Option A: Extract 0 tokens from the donation to World Food Programme

Option B: Extract 30 tokens from the donation to World Food Programme

**Remember!**

Each person in your group chooses between Option A and Option B. If this decision is selected for payment, the option chosen by you will be implemented for you. This holds for all group members: the option that each group member chose in this decision, will be implemented for themselves.

Please choose between Option A and Option B.

- Option A: Extract 0 tokens from the donation to World Food Programme
- Option B: Extract 30 tokens from the donation to World Food Programme

**PART 2**

*INSTRUCTIONS FOR BOTH INDIVIDUAL CONDITION AND VOTING CONDITION*

[NEW SCREEN]

**In the following part, you will have the possibility to earn additional money.**

You will answer two questions about what you think the other participants of your group chose in the previous questions (for all the four organisations).
 
There are two questions on each screen: 
Question A
Question B
 
Question A has a correct answer. 100 participants will be randomly selected. If you are selected you will earn an additional 5 tokens for each time your response match the true answer on Question A. (i.e. you have the possibility to earn up to 20 tokens if you answer all four questions correct).

[NEW SCREEN]

The following questions are regarding the donation to **UNICEF.**

[Picture from UNICEF]

**Question A.**

Please indicate what you think the other participants in your group chose.

How many of the other participants in your group do you believe chose Option A? (Extract 0 tokens from the donation) : _______

How many of the other participants in your group do you believe chose Option B? (Extract 30 tokens from the donation) : _______

Total : ________

**Question B.**

How likely do you think it is that 2 other group members chose Option A and that 2 other group members chose Option B?

- 1. Extremely likely
- 2.
- 3.
- 4.
- 5.
- 6. Extremely unlikely

 [NEW SCREEN]

The following questions are regarding the donation to**VI Agroforestry.**

[Picture from VI Agroforestry]

**Question A.**

Please indicate what you think the other participants in your group chose.

How many of the other participants in your group do you believe chose Option A? (Extract 0 tokens from the donation) : _______

How many of the other participants in your group do you believe chose Option B? (Extract 30 tokens from the donation) : _______

Total : ________

**Question B.**

How likely do you think it is that 2 other group members chose Option A and that 2 other group members chose Option B?

- 1. Extremely likely
- 2.
- 3.
- 4.
- 5.
- 6. Extremely unlikely

 [NEW SCREEN]

The following questions are regarding the donation to**World Child Cancer.**

[Picture from WCC]

**Question A.**

 Please indicate what you think the other participants in your group chose.

How many of the other participants in your group do you believe chose Option A? (Extract 0 tokens from the donation) : _______

How many of the other participants in your group do you believe chose Option B? (Extract 30 tokens from the donation) : _______

Total : ________

**Question B.**

 How likely do you think it is that 2 other group members chose Option A and that 2 other group members chose Option B?

- 1. Extremely likely
- 2.
- 3.
- 4.
- 5.
- 6. Extremely unlikely

 [NEW SCREEN]

The following questions are regarding the donation to**World Food Programme.**

[Picture from WFP]

**Question A.**

Please indicate what you think the other participants in your group chose.

How many of the other participants in your group do you believe chose Option A? (Extract 0 tokens from the donation) : _______

How many of the other participants in your group do you believe chose Option B? (Extract 30 tokens from the donation) : _______

Total : ________

**Question B.**

 How likely do you think it is that 2 other group members chose Option A and that 2 other group members chose Option B?

- 1. Extremely likely
- 2.
- 3.
- 4.
- 5.
- 6. Extremely unlikely

 [NEW SCREEN]

You will now be presented to some sets of questions.  There are no "right" or "wrong" answers, so please state your opinion as honestly as possible.

(Participants answered these questions on a 6-poin likert scale: Strongly disagree, Moderately disagree, Slightly disagree, Slightly agree, Moderately agree, Strongly agree)

People in our society often disagree about how far to let individuals go in making decisions for themselves.  How strongly you agree or disagree with each of these statements?

wvs1 The government interferes far too much in our everyday lives.

Rwvs2 Sometimes government needs to make laws that keep people from hurting themselves.

wvs3 It's not the government's business to try to protect people from themselves.

wvs4 The government should stop telling people how to live their lives.

Rwvs5 The government should do more to advance society's goals, even if that means limiting the freedom and choices of individuals.

Rwvs6 Government should put limits on the choices individuals can make so they don't get in the way of what's good for society.

Listed below are some characteristics that may describe a person:

Caring

Compassionate

Fair

Friendly

Generous

Helpful

Hardworking

Honest

Kind

The person with these characteristics could be you or it could be someone else. For a moment, visualize in your mind the kind of person who has these characteristics. Imagine how that person would think, feel, and act. When you have a clear image of what this person would be like, answer the following questions.

(Participants answer on a 7-point scale from Completely disagree, to Completely agree)

inter1 It would make me feel good to be a person who has these characteristics.

inter2 Being someone who has these characteristics is an important part of who I am.

inter3R I would be ashamed to be a person who has these characteristics.

inter4R Having these characteristics is not really important to me.

inter5 I strongly desire to have these characteristics.

symbol1 I often wear clothes that identify me as having these characteristics.

symbol2 The types of things I do in my spare time (e.g., hobbies) clearly identify me as having these characteristics.

symbol3 The kinds of books and magazines that I read identify me as having these characteristics.

symbol4 The fact that I have these characteristics is communicated to others by my membership in certain organisations.

symbol5 I am actively involved in activities that communicate to others that I have these characteristics.

What is your gender?

- Female
- Male

How old are you?

## Experimental instructions (Study 3)

**INSTRUCTIONS PART 1**

*MEDIAN VOTING CONDITION*

Welcome and thank you for your participation in this study!
   
**All statements made in this study are true i.e there is no deception in this study.**

During this study we will speak in terms of tokens instead of pounds (GBP). Your earnings will be calculated in terms of tokens and then translated at the end of the study into pounds (GBP) at the following rate:

**10 tokens = £ 0.5**

You will receive a participation fee (simply for completing this study). In addition, you have the possibility to gain additional earnings.

**Please note:** During the study, there will be an attention check in order to ensure that you are paying attention to the study instructions. If you fail this attention check you will be excluded from the study, and you will receive no payment.

Participation in the study should take about 15 minutes. Your decisions in this study will not be known to any other participant, i.e., your anonymity is ensured. There are no foreseeable risks to your participation in this study. By clicking on the button below, you indicate that you understand the information that was presented and that your participation is voluntary, and you may withdraw your consent and discontinue

participation in the project at any time.

- I consent to participate in this study

Please enter your Prolific ID

________________________________________________________________

[NEW SCREEN]

**Your group.**

In this study, you are together with 4 other participants from Prolific i.e. in a group of 5 people. Your group members have been allotted to you at the beginning of the study. You will at no point learn which participant is in your group.

**The donation.**

Your group is entrusted with a donation of 150 tokens designated to a charitable cause.

You and the other people in your group will all be presented with identical questions. Your own earnings and the donation depend on how you and the others respond.

**YOUR DECISION:**

Each person in your group proposes an amount to extract from the donation, between 0 and 30 tokens.

All 5 proposals are sorted from smallest to largest value.

The value in the middle is then selected: everyone in your group receives that amount as payment.

[NEW SCREEN]

Here are two examples:

**Example 1**. The five participants in a group propose to extract the following tokens:

Participant 1: 30 tokens

Participant 2: 30 tokens

Participant 3: 25 tokens

Participant 4: 15 tokens

Participant 5: 25 tokens

The values are sorted from smallest to largest (15, 25, 25, 30, 30) and the middle value is selected: 25. Participants in this group will **additionally** receive 25 tokens per person. This holds for all group members: all group members will additionally receive 25 tokens. As a further consequence, 125 tokens (25*5) of the donation of 150 tokens will be destroyed.

**Example 2.** The five participants in a group propose to extract the following tokens:

Participant 1: 0 tokens

Participant 2: 5 tokens

Participant 3: 10 tokens

Participant 4: 0 tokens

Participant 5: 10 tokens

The values are sorted from smallest to largest (0, 0, 5, 10, 10) and the middle value is selected: 5. Participants in this group will **additionally** receive 5 tokens per person at the end of the study. This holds for all group members: all group members will additionally receive 5 tokens.
 As a further consequence, 25 tokens (5*5 tokens) of the donation of 150 tokens will be destroyed.

To make sure you understand the instructions, please answer the comprehension questions below.

YOU MUST ANSWER THIS QUESTIONS CORRECTLY TO BE ELIGIBLE TO RECEIVE YOUR ADDITIONAL EARNINGS.

The five participants in a group propose to extract the following tokens:

Participant 1: 0 tokens

Participant 2: 5 tokens

Participant 3: 25 tokens

Participant 4: 30 tokens

Participant 5: 15 tokens

a) How many additional tokens will these 5 participants receive per person?

- 0 tokens
- 5 tokens
- 25 tokens
- 30 tokens
- **15 tokens**

b) How many tokens of the donation of 150 tokens will be destroyed?

- 0 tokens
- 150 tokens
- **75 tokens**
- 15 tokens
- 100 tokens

This is an attention check. This test is simple, when asked for a number to enter you must tick on the number “5” below.

Based on the text you read above, what number have you been asked to enter?

- 1
- 2
- 3
- 4
- **5**

*[Correct answers are marked with bold text in this document]*

[NEW SCREEN]

In what follows, four different charity organisations will be presented. You and the other group members will make a decision for each of the four charity organisation. At the end of the study, one of the four organisations will be randomly selected for payment, and the median value of the proposals in your group for that particular organisation will be implemented.

[NEW SCREEN]

**UNICEF**

**Measles is highly infectious and very often deadly. Each day hundreds of children become victims of this disease. The survivors often suffer consequences for their whole life, like blindness or brain damages. However, protecting the children from measles is easy. Measles vaccination offers quick, reliable, and cheap protection.**

Measles are extremely infectious and spread especially fast when many people live densely together, as in refugee camps. Especially with weakened children the disease often ends deadly or leads to lasting physical or mental damages. Measles are one of the main causes for blindness among children and often become critical when no medical help is available. UNICEF conducts major vaccination campaigns, especially after natural disasters and in other emergency situations, to prevent the spreading of the disease. With a measles vaccination you do not only protect the children, but you also reduce the risk for all who get in contact with them.

[NEW SCREEN]

**YOUR DECISION:**

Your group is entrusted with a donation totaling 150 tokens, corresponding to 30 doses of measles vaccine.

**Remember!**
Each person in your group proposes an amount to extract from the donation, between 0 and 30 tokens. All 5 proposals are sorted from smallest to largest value. If this decision is selected for payment, the value in the middle is then selected: each of you receive that amount as payment.


 How many tokens do you propose to extract from the donation to UNICEF?

________________________________________________________________

[NEW SCREEN]

**VI Agroforestry**

**Climate change is hitting hardest on poor farmers in eastern Africa – people who already live on small margins. Through tree planting and agroforestry, farmers can continue to develop their agriculture, despite drought and flooding, and the method ensures families’ access to food.**

Donations to the VI Agroforestry contributes to the planting of trees, which helps to reduce poverty and improve the environment in East Africa. Vi Agroforestry work together with small-holder farming families and farmers’ organisations in the Lake Victoria basin in East Africa. The foundation of Vi Agroforestry’s work is sustainable agriculture and agroforestry – growing trees alongside crops and livestock. It provides increased access to food, access to sustainable energy sources and more income.

[Picture from Vi Agroforestry]

[NEW SCREEN]

**YOUR DECISION:**

Your group is entrusted with a donation totaling 150 tokens, corresponding to implantation of five trees.

**Remember!**
Each person in your group proposes an amount to extract from the donation, between 0 and 30 tokens. All 5 proposals are sorted from smallest to largest value. If this decision is selected for payment, the value in the middle is then selected: each of you receive that amount as payment.

How many tokens do you propose to extract from the donation to VI Agroforestry?

________________________________________________________________

[NEW SCREEN]

**World Child Cancer

Over 300,000 children worldwide develop cancer each year. If the disease is detected early enough, many of these children can be cured -  80% of children with cancer in the high income world survive. However, in low and middle income countries, survival rates can be as low as 10%, with many children dying without effective pain relief. A reason for this inequality is that many children remain undiagnosed, or are diagnosed too late to be cured, due to a shortage of trained doctors and nurses in-country.**

World Child Cancer work to improve diagnosis, access to treatment and quality of support for children with cancer, and their families, in the low- and middle-income world.  Donations to World Child Cancer supports:

·       Chemotherapy for children with cancer
 ·       Pain relief for children with cancer
 ·       Accurate diagnosis of children with cancer.

[Picture from WCC]

[NEW SCREEN]

**YOUR DECISION:**

Your group is entrusted with a donation totaling 150 tokens, which can support the accurate diagnosis of 4 children with cancer.

**Remember!**
Each person in your group proposes an amount to extract from the donation, between 0 and 30 tokens. All 5 proposals are sorted from smallest to largest value. If this decision is selected for payment, the value in the middle is then selected: each of you receive that amount as payment.


 How many tokens do you propose to extract from the donation to World Child Cancer?

________________________________________________________________

[NEW SCREEN]

**World Food Programme**

**Some 20 million people face catastrophe in South Sudan, Somalia, Nigeria and Yemen with famine already declared in South Sudan. People are already dying from starvation and in South Sudan alone 1 million children are estimated to be acutely malnourished. If we don’t reach people with urgent food aid soon, many of them will die.**

World Food Programme (WFP) is the food-assistance branch of the United Nations and works to help people who cannot produce or obtain enough food for themselves and their families. WFP’s efforts focus on emergency assistance, relief and rehabilitation, development aid and special operations. In emergencies, WFP is often first on the scene, providing food assistance to the victims of war, civil conflict, drought, floods, earthquakes, hurricanes, crop failures and natural disasters.

[Picture from WFP]

[NEW SCREEN]

**YOUR DECISION:**

Your group is entrusted with a donation totaling 150 tokens, which represents 20 days supply of food to a hungry child.

**Remember!**
Each person in your group proposes an amount to extract from the donation, between 0 and 30 tokens. All 5 proposals are sorted from smallest to largest value. If this decision is selected for payment, the value in the middle is then selected: each of you receive that amount as payment.

 How many tokens do you propose to extract from the donation to World Food Programme?

________________________________________________________________

[NEW SCREEN]

**INSTRUCTIONS PART 1**

*INDIVIDUAL CONDITION*

Welcome and thank you for your participation in this study!

**All statements made in this study are true i.e there is no deception in this study.**

During this study we will speak in terms of tokens instead of pounds (GBP). Your earnings will be calculated in terms of tokens and then translated at the end of the study into pounds (GBP) at the following rate:

**10 tokens = £ 0.5**

You will receive a participation fee (simply for completing this study). In addition, you have the possibility to gain additional earnings.

**Please note:** During the study, there will be an attention check in order to ensure that you are paying attention to the study instructions. If you fail this attention check you will be excluded from the study, and you will receive no payment.

Participation in the study should take about 15 minutes. Your decisions in this study will not be known to any other participant, i.e., your anonymity is ensured. There are no foreseeable risks to your participation in this study. By clicking on the button below, you indicate that you understand the information that was presented and that your participation is voluntary, and you may withdraw your consent and discontinue participation in the project at any time.

- I consent to participate in this study

Please enter your Prolific ID

________________________________________________________________

**Your group.**

In this study, you are together with 4 other participants from Prolific i.e. in a group of 5 people. Your group members have been allotted to you at the beginning of the study. You will at no point learn which participant is in your group.

**The donation.**

Your group is entrusted with a donation of 150 tokens designated to a charitable cause. You and the other people in your group will all be presented with identical questions. Your own earnings and the donation depend on how you respond.

**YOUR DECISION**:

Each person in your group chooses an amount to extract from the donation, between 0 and 30 tokens.

Everyone in your group receives the amount they choose to extract as payment.

[NEW SCREEN]

Here are two examples:

**Example 1.** The five participants in a group extract the following tokens:

Participant 1: 30 tokens

Participant 2: 30 tokens

Participant 3: 25 tokens

Participant 4: 15 tokens

Participant 5: 25 tokens

Together the participants extracted 30 + 30 + 25 + 15 + 25= 125 tokens. Each person receives the amount they extract as payment. For example, participant 5 will **additionally** receive 25 tokens. This holds for all group members: all group members will additionally receive the amount they extract.

As a further consequence, 125 tokens (30 + 30 + 25 + 15 + 25) of the donation of 150 tokens will be destroyed

**Example 2.** The five participants in a group extract the following tokens:

Participant 1: 0 tokens

Participant 2: 10 tokens

Participant 3: 10 tokens

Participant 4: 0 tokens

Participant 5: 5 tokens

Together the participants extracted 0 + 10 + 10 + 0 + 5= 25 tokens. For example, participant 5 will **additionally** receive 5 tokens at the end of the study. This holds for all group members: all group members will additionally receive the amount they extract. As a further consequence, 25 tokens (0 + 10 + 10 + 0 + 5) of the donation of 150 tokens will be destroyed.

To make sure you understand the instructions, please answer the comprehension questions below.

YOU MUST ANSWER THIS QUESTIONS CORRECTLY TO BE ELIGIBLE TO RECEIVE YOUR ADDITIONAL EARNINGS.

The five participants in a group propose to extract the following tokens:

Participant 1: 0 tokens

Participant 2: 5 tokens

Participant 3: 25 tokens

Participant 4: 30 tokens

Participant 5: 15 tokens

a) How many additional tokens will participant 5 receive?

o 0 tokens

o 5 tokens

o 25 tokens

o 30 tokens

**o 15 tokens**

b) How many tokens of the donation of 150 tokens will be destroyed?

o 0 tokens

o 150 tokens

**o 75 tokens**

o 15 tokens

o 100 tokens

This is an attention check. This test is simple, when asked for a number to enter you must tick on the number “5” below.

Based on the text you read above, what number have you been asked to enter?

o 1

o 2

o 3

o 4

**o 5**

*[Correct answers are marked with bold text in this document]*

[NEW SCREEN]

In what follows, four different charity organisations will be presented. You and the other group members will make a decision for each of the four charity organisation. At the end of the study, one of the four organisations will be randomly selected for payment, and the value that you chose for that particular organisation will be implemented. The other group members will also have their decision implemented for that particular organisation.

[NEW SCREEN]

**UNICEF**

**Measles is highly infectious and very often deadly. Each day hundreds of children become victims of this disease. The survivors often suffer consequences for their whole life, like blindness or brain damages. However, protecting the children from measles is easy. Measles vaccination offers quick, reliable, and cheap protection.**

Measles are extremely infectious and spread especially fast when many people live densely together, as in refugee camps. Especially with weakened children the disease often ends deadly or leads to lasting physical or mental damages. Measles are one of the main causes for blindness among children and often become critical when no medical help is available. UNICEF conducts major vaccination campaigns, especially after natural disasters and in other emergency situations, to prevent the spreading of the disease. With a measles vaccination you do not only protect the children, but you also reduce the risk for all who get in contact with them.

[Picture from UNICEF]

[NEW SCREEN]

**YOUR DECISION:**

Your group is entrusted with a donation totaling 150 tokens, corresponding to 30 doses of measles vaccine.

**Remember!**

Each person in your group choose an amount to extract from the donation, between 0 and 30 tokens. If this decision is selected for payment, you receive the amount you choose to extract as payment.

How many tokens do you want to extract from the donation to UNICEF?

________________________________________________________________

[NEW SCREEN]

**VI Agroforestry**

**Climate change is hitting hardest on poor farmers in eastern Africa - people who already live on small margins. Through tree planting and agroforestry, farmers can continue to develop their agriculture, despite drought and flooding, and the method ensures families' access to food.**

Donations to the VI Agroforestry contributes to the planting of trees, which helps to reduce poverty and improve the environment in East Africa. Vi Agroforestry work together with small-holder farming families and farmers’ organisations in the Lake Victoria basin in East Africa. The foundation of Vi Agroforestry’s work is sustainable agriculture and agroforestry – growing trees alongside crops and livestock. It provides increased access to food, access to sustainable energy sources and more income.

[Picture from VI Agroforestry]

[NEW SCREEN]

**YOUR DECISION:**

Your group is entrusted with a donation totalling 150 tokens, corresponding to implantation of five trees.

**Remember!** Each person in your group choose an amount to extract from the donation, between 0 and 30 tokens. If this decision is selected for payment, you receive the amount you choose to extract as payment.

How many tokens do you want to extract from the donation to VI Agroforestry?

________________________________________________________________

[NEW SCREEN]

**World Child Cancer**

**Over 300,000 children worldwide develop cancer each year. If the disease is detected early enough, many of these children can be cured -  80% of children with cancer in the high income world survive. However, in low and middle income countries, survival rates can be as low as 10%, with many children dying without effective pain relief. A reason for this inequality is that many children remain undiagnosed, or are diagnosed too late to be cured, due to a shortage of trained doctors and nurses in-country.**

World Child Cancer work to improve diagnosis, access to treatment and quality of support for children with cancer, and their families, in the low- and middle-income world.  Donations to World Child Cancer supports:

- Chemotherapy for children with cancer
- Pain relief for children with cancer
- Accurate diagnosis of children with cancer

[Picture from WCC]

[NEW SCREEN]

**YOUR DECISION:**

Your group is entrusted with a donation totaling 150 tokens,  corresponding to accurate diagnosis of 4 children with cancer

**Remember!**
Each person in your group choose an amount to extract from the donation, between 0 and 30 tokens. If this decision is selected for payment, you receive the amount you choose to extract as payment.

How many tokens do you want to extract from the donation to World Child Cancer? ________________________________________________________________

[NEW SCREEN]

**World Food Programme**

**Some 20 million people face catastrophe in South Sudan, Somalia, Nigeria and Yemen with famine already declared in South Sudan. People are already dying from starvation and in South Sudan alone 1 million children are estimated to be acutely malnourished. If we don’t reach people with urgent food aid soon, many of them will die.**

World Food Programme (WFP) is the food-assistance branch of the United Nations and works to help people who cannot produce or obtain enough food for themselves and their families. WFP’s efforts focus on emergency assistance, relief and rehabilitation, development aid and special operations. In emergencies, WFP is often first on the scene, providing food assistance to the victims of war, civil conflict, drought, floods, earthquakes, hurricanes, crop failures and natural disasters.

[Picture from WFP]

[NEW SCREEN]

**YOUR DECISION:**

Your group is entrusted with a donation totaling 150 tokens, which represents 20 days supply of food to a hungry child.

**Remember!** Each person in your group choose an amount to extract from the donation, between 0 and 30 tokens. If this decision is selected for payment, you receive the amount you choose to extract as payment.

How many tokens do you want to extract from the donation to World Food Programme?

________________________________________________________________

**PART 2**

*INSTRUCTIONS FOR BOTH MEDIAN VOTING CONDITING AND INDIVIDUAL CONDITION*

**In the following part, you will have the possibility to earn additional money.**
 
You will answer two questions about what you think the other participants of your group chose in the previous questions (for all the four organisations).
 
There are two questions on each screen: 
Question A
Question B
 
Question A has a correct answer. 100 participants will be randomly selected. If you are selected you will earn an additional 5 tokens for each time your response match the true answer on Question A. (i.e. you have the possibility to earn up to 20 tokens if you answer all four questions correct).

[NEW SCREEN]

The following questions are regarding the donation to **UNICEF.**

[Picture from UNICEF]

**Question A.**

Please indicate what you think the other participants in your group chose.

How many participants in your group do you believe proposed to extract **more** from the donation to than you? : _______ (1)

How many participants in your group do you believe proposed to extract **less** from the donation than you? : _______ (2)

How many participants in your group do you believe proposed to extract **the same amount** from the donation as you? : _______ (3)

Total : ________

**Question B.**

How likely do you think it is that the value that you proposed to extract from the donation to UNICEF was the middle value of all five proposals in your group?

- Extremely likely
- 2.
- 3.
- 4.
- 5.
- 6. Extremely unlikely

[NEW SCREEN]

The following questions are regarding the donation to**VI Agroforestry.**

[Picture from VI Agroforestry]

**Question A.**

Please indicate what you think the other participants in your group chose.

How many participants in your group do you believe proposed to extract  **more** from the donation to than you? : _______

How many participants in your group do you believe proposed to extract **less** from the donation than you? : _______

How many participants in your group do you believe proposed to extract **the same amount** from the donation as you? : _______

Total : ________

**Question B.**

 How likely do you think it is that the value that you proposed to extract from the donation to VI Agroforestry was the middle value of all five proposals in your group?

- 1. Extremely likely
- 2.
- 3.
- 4.
- 5.
- 6. Extremely unlikely

[NEW SCREEN]

The following questions are regarding the donation to**World Child Cancer.**

[Picture from WCC]

**Question A.**
Please indicate what you think the other participants in your group chose.

How many participants in your group do you believe proposed to extract  **more** from the donation to than you? : _______ (1)

How many participants in your group do you believe proposed to extract **less** from the donation than you? : _______ (2)

How many participants in your group do you believe proposed to extract **the same amount** from the donation as you? : _______ (3)

Total : ________

**Question B.**
How likely do you think it is that the value that you proposed to extract from the donation to World Child Cancer was the middle value of all five proposals in your group?

- 1. Extremely likely
- 2.
- 3.
- 4.
- 5.
- 6. Extremely unlikely

[NEW SCREEN]

The following questions are regarding the donation to**World Food Programme.**

[Picture from WFP]

**Question A.**
Please indicate what you think the other participants in your group chose.

How many participants in your group do you believe proposed to extract  **more** from the donation to than you? : _______

How many participants in your group do you believe proposed to extract **less** from the donation than you? : _______

How many participants in your group do you believe proposed to extract **the same amount** from the donation as you? : _______

Total : ________

**Question B.**

How likely do you think it is that the value that you proposed to extract from the donation to World Food Programme was the middle value of all five proposals in your group?

- 1. Extremely likely
- 2.
- 3.
- 4.
- 5.
- 6. Extremely unlikely

[NEW SCREEN]

You will now be presented to some sets of questions.  There are no "right" or "wrong" answers, so please state your opinion as honestly as possible.

(Participants answered these questions on a 6-poin likert scale: Strongly disagree, Moderately disagree, Slightly disagree, Slightly agree, Moderately agree, Strongly agree)

People in our society often disagree about how far to let individuals go in making decisions for themselves.  How strongly you agree or disagree with each of these statements?

wvs1 The government interferes far too much in our everyday lives.

Rwvs2 Sometimes government needs to make laws that keep people from hurting themselves.

wvs3 It's not the government's business to try to protect people from themselves.

wvs4 The government should stop telling people how to live their lives.

Rwvs5 The government should do more to advance society's goals, even if that means limiting the freedom and choices of individuals.

Rwvs6 Government should put limits on the choices individuals can make so they don't get in the way of what's good for society.

Listed below are some characteristics that may describe a person:

Caring

Compassionate

Fair

Friendly

Generous

Helpful

Hardworking

Honest

Kind

The person with these characteristics could be you or it could be someone else. For a moment, visualize in your mind the kind of person who has these characteristics. Imagine how that person would think, feel, and act. When you have a clear image of what this person would be like, answer the following questions.

(Participants answer on a 7-point scale from Completely disagree, to Completely agree)

inter1 It would make me feel good to be a person who has these characteristics.

inter2 Being someone who has these characteristics is an important part of who I am.

inter3R I would be ashamed to be a person who has these characteristics.

inter4R Having these characteristics is not really important to me.

inter5 I strongly desire to have these characteristics.

symbol1 I often wear clothes that identify me as having these characteristics.

symbol2 The types of things I do in my spare time (e.g., hobbies) clearly identify me as having these characteristics.

symbol3 The kinds of books and magazines that I read identify me as having these characteristics.

symbol4 The fact that I have these characteristics is communicated to others by my membership in certain organisations.

symbol5 I am actively involved in activities that communicate to others that I have these characteristics.

What is your gender?

- Female
- Male

How old are you?
